# Young Adults Journey with Digital Fitness Tools-A Qualitative Study on Use of Fitness Tracking Device

**DOI:** 10.12688/f1000research.158037.1

**Published:** 2024-10-29

**Authors:** Asees Kaur Gulati, Rachel Edna Lobo, Nihala N, Vishweshwara Bhat, Neha Bora, Vaishali K, Mukesh Kumar Sinha

**Affiliations:** 1 Department of Physiotherapy, Manipal College of Health Professions, Manipal Academy of Higher Education, Manipal, Karnataka, 576104, India

**Keywords:** Digital tracking, wearables, mobile health, physical activity, exercise, device, young adults, barriers and facilitators, fitness

## Abstract

**Background:**

Physical activity trackers possess the potential to encourage increased physical activity. However, users often exhibit poor long-term adherence to these devices, which may stem from a lack of understanding of the factors influencing their use, as well as changes in health behavior. This study aims to provide new insights into the types of health-related apps and devices that young people discover, select, and utilize, along with the underlying reasons for their choices.

**Methods:**

Young adults aged between 18-25 years reporting the use and nonuse of health apps and wearables were recruited and participated in focus group discussions about app choice, features, their perceptions towards the physical activity trackers, and reasons for continued use and for not using one. A total of Nine FGDS were conducted among users, nonusers, and former users of physical activity tracker usage. Data was recorded, transcribed, and analyzed for themes in an iterative approach.

**Results:**

The physical activity tracker users group stated that they had a sense of achievement, validation, and other motivational feedback, which helped with adherence. All three groups (user, non-user, and former user) had similar concerns about cost, reliability, accuracy, and dependency. Former users also reported that the idea of accomplishing the goals would create anxiety and, in turn, harm the body. Former users and nonusers expressed their concerns regarding lack of time and loss of motivation to track their physical activity. Nonusers had issues with the functionality of the device (ease of use, battery, notifications, apps hang) and were also aware of all the health benefits of using wearables.

**Conclusion:**

Physical activity users group, former users group, and nonusers group shared concerns about accuracy, cost, and battery.

## Introduction

Engaging in regular physical activity is crucial for reducing the risk of noncommunicable diseases, maintaining both physical and cognitive functions, and improving overall well-being. ^1^ Despite this, data from 2022 shows that about one-third of adults worldwide, roughly 1.8 billion people, did not meet the recommended activity levels. ^2^ Insufficient physical activity is linked to a greater risk of various health issues, including major non-communicable diseases like coronary heart disease, type 2 diabetes, breast cancer, and colon cancer. ^3^ It also leads to reduced life expectancy, making it a significant public health concern. ^4^ Although numerous policies have been introduced to boost physical activity globally, there has been little progress in raising activity levels among adults. This situation underscores the need for renewed efforts and innovative methods to effectively promote physical activity and encourage a more active lifestyle across populations worldwide.

In today’s increasingly digital world, the integration of technology into daily life has revolutionized many areas, including fitness and health. Digital fitness tools, such as apps, wearables, and online platforms, have gained substantial popularity, especially among young adults. ^5– 7^ This demographic, known for being tech-savvy and highly focused on personal wellness, is adopting these innovations to increase physical activity, track fitness goals, and lead healthier lives. Wearable activity trackers, which are becoming more common globally, offer significant potential for supporting physical activity among a broad audience. ^5– 7^ Young people, in particular, are keen on finding, selecting, and utilizing mobile digital applications. These wearables enable users to monitor real-time physical activity, often using accelerometers, energy expenditure, heart rate, and other sensors.

Additionally, young adults are more likely than any other age group to use their phones for health-related information. Previous studies indicate that college students spend an average of 279 to 528 minutes per day using their devices with those who use their devices most frequently engaging with them nearly all the time. ^8– 10^ The use of cell phones is a prevalent behavior among individuals of various age groups. Wearables are defined as any body-worn computer designed to offer useful services while the user performs other tasks. These include activity trackers, pedometers, accelerometers, smartwatches, or smart clothing, which can be used to self-monitor and thereby improve physical activity levels and reduce sedentary behavior. Self-monitoring of activity levels has been associated with increased physical activity. ^6^

However, long-term adherence to using these tools remains limited. Moreover, the rise of digital fitness tools, or Fitness tracking device, is changing how young adults approach health and wellness. Despite their growing popularity and accessibility, there’s a lack of understanding of the deeper, experiential impact of these tools on users’ fitness journeys. Current research mostly quantifies usage patterns and general health outcomes but lacks qualitative insights into young adults’ personal experiences, challenges, and successes with these tools. Understanding these aspects can provide valuable insights for developers, healthcare professionals, and educators to enhance fitness-tracking device design and implementation, fostering more supportive environments for young adults to meet their fitness goals. This study aims to address this gap by exploring the unique stories of young adults using digital fitness tools, thereby contributing to a better understanding of their role in modern fitness culture.

## Methods

### Design

This study employs Focus Group Discussions (FGD) as a qualitative research method to delve into young adults’ distinct perceptions of digital fitness tools. By engaging participants in guided group discussions, the study explores how these individuals perceive and interact with technologies such as fitness apps and wearable devices. The objective is to gain deeper insights into their attitudes, motivations, and the impact of these tools on their fitness routines. Additionally, this manuscript adheres to the COREQ (Consolidated Criteria for Reporting Qualitative Research) checklist, ensuring transparency and comprehensiveness in the reporting of the study’s methodology and findings. ^11^

### Participant selection

Young, healthy individuals aged 18 to 25 years of both genders were selected to participate. The participants were divided into three groups: current users of physical activity trackers (apps or wearables), former users who had stopped using these trackers, and nonusers who had never used them. Differently, abled participants were excluded.

Using a purposive sampling technique, the study employed a saturation sampling approach. This qualitative sampling method involves recruiting participants until no new information is obtained, supporting the emergent nature of qualitative research and enabling simultaneous data collection and analysis. Separate focus group discussions were held for each group to encourage open and candid discussions about their perceptions of physical activity trackers. Individuals with recent injuries, fractures, surgeries, cardiopulmonary or metabolic issues, neurological complaints, or difficulties comprehending were excluded from the study. A brief demographic data was collected to capture basic information about participants before the FGD. They were categorized into three groups after screening, and written informed consent was obtained from each participant before the focus group discussion. This process includes outlining the study’s objectives, the criteria for participation, and any possible risks. Participants were assured of their confidentiality and the voluntary nature of their involvement, meaning they were free to withdraw at any time without consequence.

### Interview guide development

An interview guide was developed following an extensive literature search using various databases like

Medline via PubMed, Scopus, Web of Science, and Embase. Face validation was carried out with eight members, including two physiotherapists involved in promoting physical activity, two exercise and sports science experts, two clinical psychologists, and two public health experts. The interview guide was modified based on the suggestions made by the expert panel members, and a final interview guide was prepared, which was used for conducting FGD. It consisted of open-ended questions and prompts eliciting rich narratives and insights from participants regarding the use of an activity tracker for physical activity.

The details of the moderator guide:

Participants were asked about their general thoughts on engaging in physical activity and exploring individual motivations and barriers. Discussions then shifted to participants’ opinions on tracking physical activity, evaluating the usefulness and relevance of monitoring habits, and their attitudes toward activity trackers.

For the non-user group, participants were asked to mention barriers preventing them from acquiring activity trackers, such as perceived cost, privacy concerns, relevance, and any other matters. They were also asked about their perception of physical activity.

For the user group, participants were asked to reflect on their experiences with activity trackers, discussing usability, effectiveness, and impacts on daily routines. They were asked to mention challenges faced while using the trackers and potential motivators for device/tracker use. They were also asked about their perceptions or beliefs regarding the use of these activity trackers.

For the former user group, participants were asked about their past experiences with activity trackers, including usage patterns and effectiveness. They were asked for reasons for discontinuing use, such as loss of interest or technical issues, and to share their feelings after stopping. Finally, they were asked what changes in wearable technology might encourage them to start using activity trackers again. Key questions for each group are depicted in supplementary Table. ^16^

### Data collection

Participants who met the inclusion criteria were invited to take part in Focus Group Discussions (FGDs) at their convenience. The FGDs were led by three female physiotherapist researchers: AKG, REL, and NN, all of whom had received training in qualitative studies. Each moderator conducted discussions with different groups. Upon arrival, participants provided informed consent and demographic details ( Table 1). The FGDs explored participants’ physical activity and experiences with wearable fitness trackers or other fitness apps. The nonuser group also discussed their knowledge and attitude towards using activity trackers. The FGDs, lasting between 45 to 90 minutes, involved a total of 56 participants and were divided into three categories: active users, non-users, and former users, with 5 to 8 participants in each group. Probes guided the discussions, which were audiotaped with participants’ consent, and supplemented by field notes. The interviews were recorded using handheld recorders (AE mini digital voice recorder, MP3 player compatible with 32GB extended TF card), and verbatim transcription was carried out.

**Table 1.** Participant characteristics.

	Users (n=14)	Former users (n=16)	Nonusers (n=26)
**Characteristics**			
Age (Years)	22.6±1	22.6±1.55	22.3±1.64
Gender	F=9, M=4	F=13, M=3	F=11, M=15
Weight (kg)	60.4±14.4	56.4±8.37	61.2 ±8.97
Height (cm)	162±9.05	165±7.67	169±6.89
BMI	23.1±5.49	20.7±2.02	21.5±2.06
**Type of wearable activity tracker**			
Samsung health	2	4	-
Apple fitness app	2	-	-
Fast track app	1	1	-
Huawei smart watch	2	1	-
Google fit	2	4	-
Step set go	4	2	-
Fossil smart watch	1	-	-
Da Fit	-	2	-
BoAt Wave	-	2	-

### Data analysis

The data was transcribed and analyzed using MS Excel. An iterative approach was employed for data analysis. The interview was coded in Microsoft Excel, and inductive content analysis was conducted. Codes were clustered into minor categories, which were further grouped into major categories. Thematic analysis was then carried out, wherein the raw data was thoroughly examined to derive themes without any restrictions.

Data analysis commenced after transcribing the first focus group discussion (FGD). Each transcript was meticulously reviewed, and each participant’s experience was identified and coded. Text segments were categorized, and relevant quotes were highlighted. Additionally, every transcript and corresponding field notes were analyzed, and new categories were added. At the same time, previously coded transcripts were checked for the presence of these new categories.

Three researchers (NN, AKG, and REL) independently derived themes and analyzed all nine FGDs. while another group of researchers (MKS and NB) analyzed every second FGD. Once no new information was obtained from two consecutive FGDs and the primary analytic structure was considered stable, final verification was carried out by another two researchers (VK and VB). The research team discussed the categories and defined emerging themes and subthemes. The generated themes were cross-referenced with the field notes and original text to accurately represent the participants’ perceptions.

## Results

**Figure 1.**
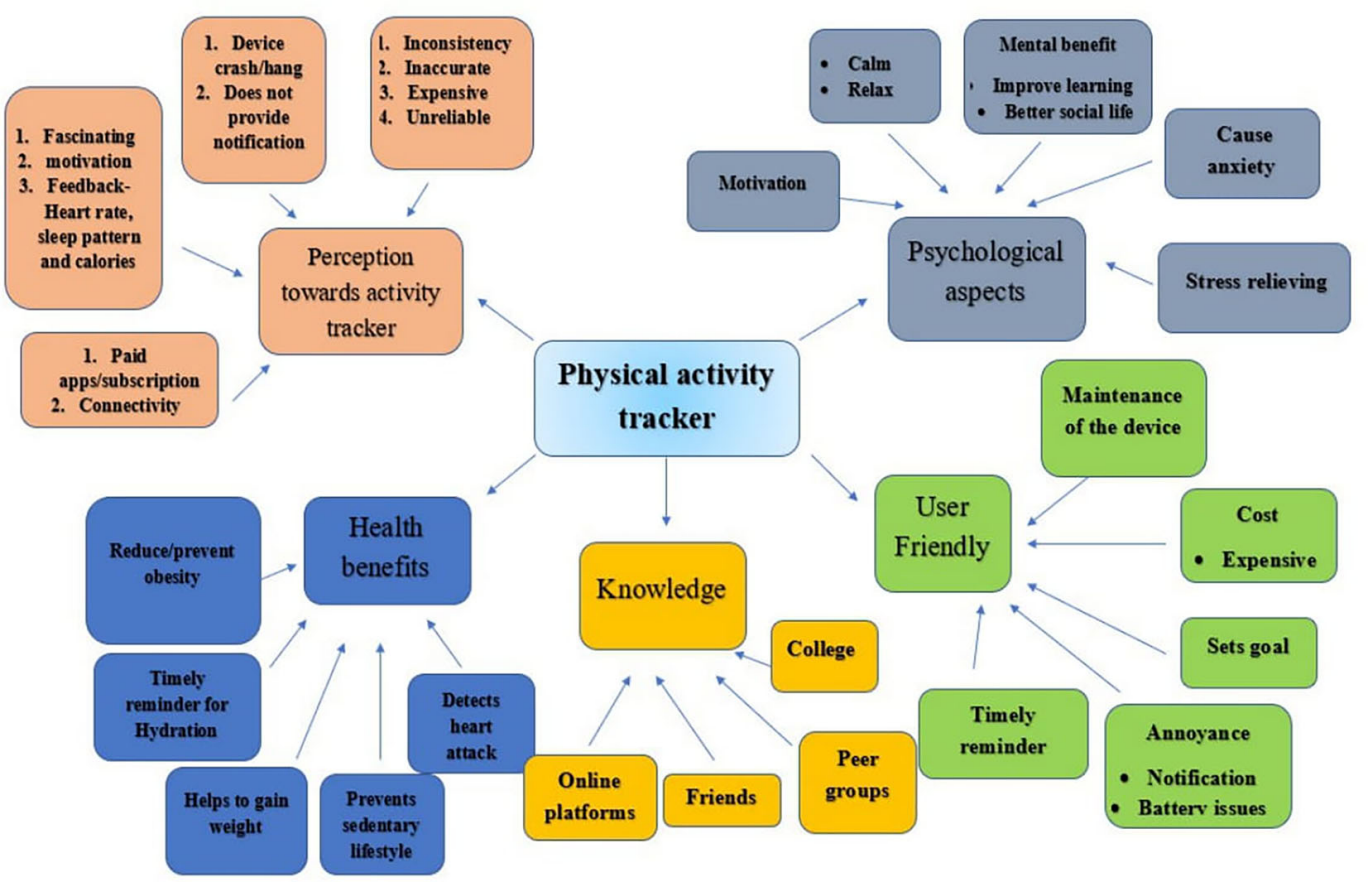
Thematic map showing Theme and code.

**Figure 2.**
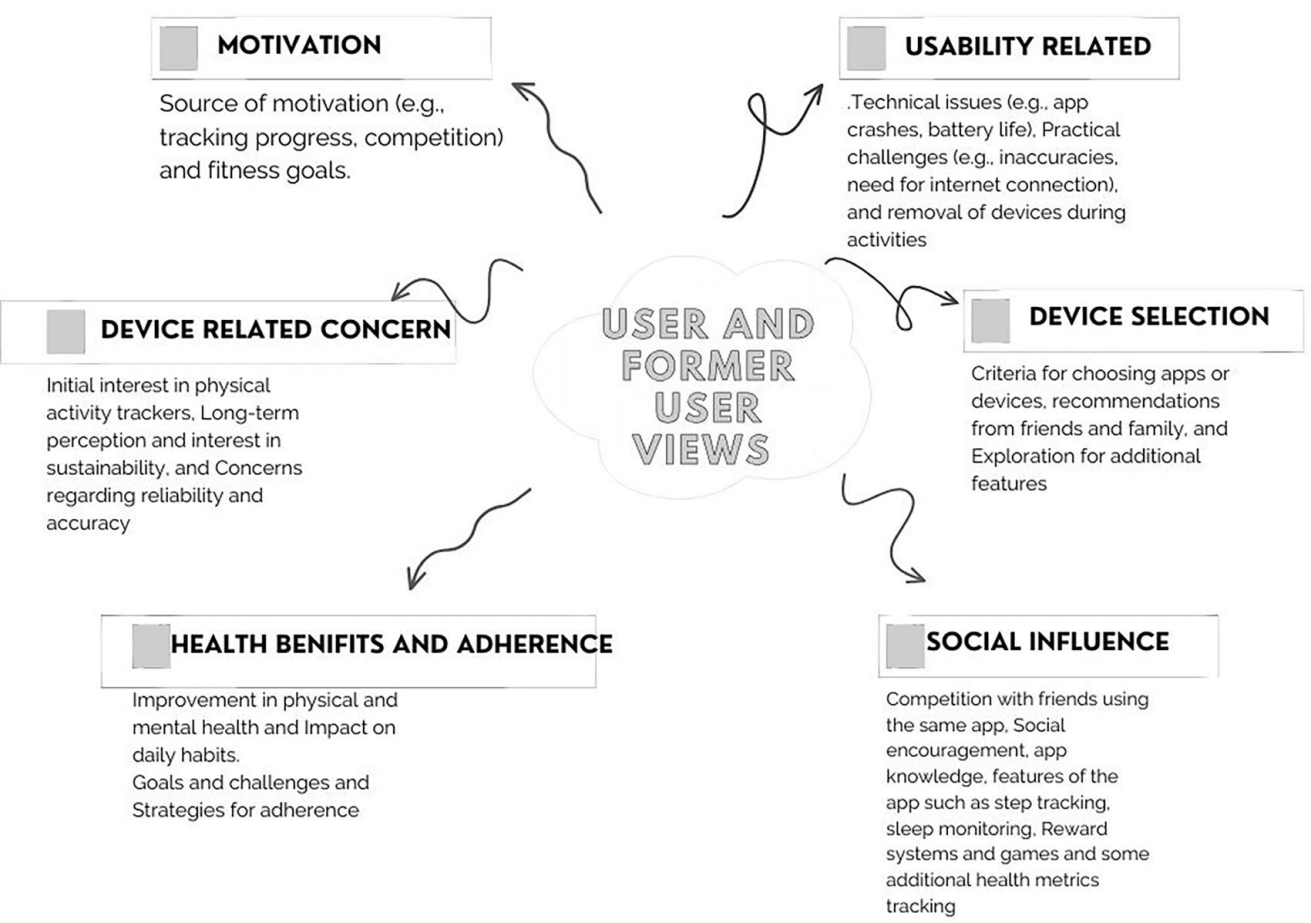
User and former user group perception related to activity tracking.

The participants’ characteristics are displayed in Table 1. A total of 9 FGDs were conducted for users, non-users, and former users. 2 FGD were conducted for the active user group (n=14), 3 FGD for the non-user group (n=26), and 4 FGD for the former user group (n=16). The participants reported using a wide range of apps, including Step Set Go, Samsung Health, Apple Fitness app, Google Fitbit, and Huawei Smartwatch ( Table 1). The analysis of the interviews revealed numerous themes related to obtaining the app, using the app, and likes and dislikes about the app. The following five main themes contributed to perceptions of physical activity trackers, psychological aspects related to physical activity trackers, user-friendliness, health benefits of physical activity, and knowledge about physical activity post tracker usage. ( Figure 1). A summary of the themes and codes is provided in Figures 2. Also, the details of the reporting from users, former users, and nonusers are depicted in Figure 2 and Table 2.

**Table 2.** Non user group viewpoints about activity tracker.

▪ **Knowledge about Physical activity-** Stress relief, physical activity is a means to release stress, provide sleep benefits, improve daily efficiency, and healthy lifestyle. ▪ Perceived need for tracking- Belief that daily activities and routines provide enough physical activity without the need for tracking, Satisfaction with current activity levels through daily chores, college, outdoor games, etc. ▪ **Opinions on Activity Trackers-** Considered a waste of time and financially unnecessary, concerns about becoming overly reliant on technology, preference for maintaining natural daily routines, desire to minimize technology use, the belief from the family. ▪ **Barriers to Adoption-** Perceived as not necessary, perceived as a financial burden, perceived as not worth the effort, about increased exposure to radiation perception, about the accuracy issue, Inability to use while sick

### Perceptions towards physical activity wearable trackers

Participants who track their physical activity have a positive perception of these trackers, which motivates them to maintain consistency, specifically the objective feedback, e.g., steps, heart rate, calorie intake, sleep pattern, exercise intensity, water intake, and electrolyte balance. This made them feel confident about their physical activity as they could see the results and progress over time.

*‘I found it really motivational and surprisingly am not a consistent person in terms of exercise, but it has actually made me like a little more consistent towards at least walking or jogging or doing some physical activity everyday’*

Despite the positive feedback, there were some issues that wearable user’s group and wearable former users group mentioned, such as the app not providing enough notifications, the app hanging and crashes, accuracy issues, draining a lot of battery from the phone since the wearable is connected to the phone- which are not enough to encourage long time usage of this devices.

*‘If my smartwatch is working, then my app won’t be monitoring accurately.’*

*‘Sometimes the apps hang, and they don’t show proper notifications or the steps, it happened to me today so you know the one thing I would like to change they should be consistent every day and it should show accurate results’*

All three groups were asked to describe aspects of the device that they would like to change or are preventing them from making a purchase. Participants in all the groups mentioned that the cost may not be worth the benefits. They were also concerned that the devices were inaccurate based on their experiences.

The Wearable nonusers group reported that the trackers are unreliable and expensive, and since there is inconsistency in them doing physical activity, they don’t see the worth of getting one physical activity tracker.

*‘I need to first start getting very regular with my physical activity cause then it will just feel like a waste of money as some of them can be quite pricey also’*

Participants in the wearables nonusers and former users group also expressed their concerns regarding the Internet connectivity that connects the wearable to the app in the phone. Some expressed that they forget to turn on their internet or are in no network range, leading to data inaccuracy.

*‘These apps require Internet connection. So sometimes we are in the area where we are not getting proper Internet connection and sometimes, we are there in that area for longer duration which might also hinder the effectiveness of tracking’*

### Psychological aspects related to physical activity tracking

Physical activity wearable users discussed their positive attitudes and emotions towards their devices. Most felt like the device created a sense of achievement by earning rewards or points after completing a goal, which kept them motivated to keep exercising.

*‘Now my watch keeps telling me get up and move’*

*‘Right now, I set it for 40 points but there is specific score which is 150 hard points for a week, and I always score above it and I always feel good about it. So, it’s more like a motivator for me to hit more than 150 points’*

The physical activity tracker users group also described a sense of achievement when competing with their peers, which keeps them motivated to reach their goals.

*‘Also, within the app, you’re able to compete with other friends or other people so that also is kind of gives you a sense of fulfillment when you see your name with the number of steps you have and how much more it is compared to your friends’*

One of the former physical activity tracker users also reported that the idea of accomplishing the goals would create anxiety and in turn harm the body

*‘I think that how much ever I eat, I should burn it or else it will cause harm to my body. I am quite particular in that way and then this creates anxiety in me that is why I am not into tracking’*

### User-friendliness

Physical activity tracking users group enjoyed using the trackers more than the non-users group. However, users wore different brands of devices, leading to discrepancies in responses. Users group reported that the notifications sent through the tracker kept them motivated to keep exercising and accomplish their goal for the day.

*‘Now my watch keeps telling me get up and move’*

*‘The app seems to give timely reminders that these many steps are remaining. Try your best, keep it up, finish it for the day.’*

All the three groups expressed their annoyance with the functionality of the app connected with the tracker.

*‘Sometimes the apps hang, and they don’t show proper notifications or the steps. It’s happened to me today. So, you know, that’s one thing I would like to change they should be consistent every day and it should show accurate results’*

Based on their personal experience, both users and non-users group expressed concerns regarding the device’s accuracy.

*‘I use a watch, but I am not sure whether what they are telling me is correct or not because, some days I feel that I work more, but when I check the tracker, I see it’s 8000 steps but I feel like I’ve walked 10,000 steps so I am not sure whether it is reliable or not’*

All the groups expressed a concern with the devices overestimating the amount of activity

*‘If you are like in a moving car or a bike, sometimes these steps add on even if you are not doing the physical activity’*

The nonusers group also had a problem with the maintenance of the device.

*‘Maintenance of these equipment’s will also be something I consider. For example, if I get a smart watch, it will be more expensive in comparison, and then I will have to take care of it.’*

Former users group expressed concerns that led them to stop using the physical activity tracker. Some did not have the time because of their professional workload, some expressed annoyance with the charging issue, and some lost motivation over time as they kept forgetting to wear their wearables.

### Health benefits

Wearable users and former users group described a certain behavior change after tracking their physical activity. They were more aware of their physical health and work more towards it. They have reported being more adherent to things in life.

*‘We are adherent to that 10,000 or 11,000 steps that adherence will help us like little more physically active than what we were previously with’*

In this study, we explore the hypothesis that the demographic composition of our participants significantly shapes the inclination towards user-friendly interfaces in health monitoring devices. Specifically, we consider variables such as age, height, weight, body mass index (BMI), and fitness tracker usage habits. Our cohort predominantly comprises younger individuals whose engagement with technology offers valuable insights into device interaction preferences. Age plays a key role in how people engage with technology, with younger users generally being more receptive to digital interfaces. Understanding age distribution helps tailor device usability for better engagement and satisfaction. Incorporating anthropometric metrics enables personalized device recommendations that align with individual health profiles. Analyzing users’ interactions with fitness trackers—active, discontinued, or non-users—provides insights into the factors that encourage or hinder consistent usage. This information is essential for designing interfaces that effectively meet user needs and overcome challenges with fitness trackers. The aspiration to achieve aesthetic appeal and sustain a healthy physique acts as a catalyzing factor, fostering increased engagement in physical activities across diverse platforms and modalities. This alignment of digital integration with fitness aspirations elucidates a synergistic relationship that enhances the overall enthusiasm toward physical activity participation.

Nonusers group have also recognized the potential of these physical activity trackers and encouraged to do physical activity as it prevents diseases.

*‘I heard that some people saved their own lives because they had some kind of a tracking device on them like the Apple version. Like one person was saved from having a cardiac arrest and it also helps people who are struggling with some physical condition like maybe diabetes or heart problem’*

Some have also expressed concerns that youngsters get deeply involved in tracking that can lead to misusing of it which might then affect their mental and physical health.

### Knowledge on physical activity

Both physical activity tracker users and non-users group expressed positive opinions on physical activity. The most common ones stated were that it is a state of physical as well as mental well-being that improves one’s quality of life and helps to prevent diseases. Many of them also reported that it brings a sense of peace and helps to relieve stress, gives a calming effect, and gives a sense of peace to mind and body, which improves their overall wellness.

The participants were asked about their source of knowledge on physical activity. Some expressed that they learned about it from online platforms. Some learnt about it from their peer groups.

## Discussion

This qualitative study delves into the experiences of young adults using digital fitness tools. The study identifies various factors from the perspectives of users, former users and nonusers of digital fitness trackers. The findings from this study provide valuable insights into how young adults utilize digital fitness tools. As the use of these technologies becomes more prevalent, it is crucial to understand their impact on health behaviors and lifestyle choices among young people.

Participants reported that digital fitness tools significantly enhance their motivation to engage in physical activity. The ability to track progress in real-time, set personalized goals, and receive immediate feedback were highlighted as key motivators that help them stay accountable. This aligns with existing literature, suggesting that digital fitness apps’ self-monitoring features can improve adherence to exercise regimens. ^12, 13^

Furthermore, social connectivity was an important theme in the discussions. Many participants expressed that the social features in these tools, such as sharing achievements with friends and participating in virtual challenges, keep motivation on the higher side, and user engagement is enhanced. This finding is consistent with previous research. ^14^

However, the study also uncovered challenges associated with the use of activity trackers. Participants indicated varying levels of technological proficiency, which influenced their overall experience with the trackers. More tech-savvy individuals could leverage advanced features to enhance their fitness journey, while others felt overwhelmed or frustrated by the technology. This difference underscores the importance of digital fitness tools with user-friendly interfaces catering to different levels of technological literacy.

## Conclusion

Our findings help to understand the characteristics that appeal to physical activity tracker users and the viewpoints of non-users and former users. All three groups expressed concerns about these devices’ accuracy, cost, and battery life. Non-users’ perspectives mainly focused on functionality, materialism, and differences in motivation compared to users. These varied perceptions of active users, non-users, and former users towards wearables will aid future physical activity research and interventions related to digital behavior.

### Ethics and consent

This study was approved by the Institutional Ethics Committee of Kasturba Medical College and Kasturba Hospital Manipal Institutional Ethics Committee-2 (Student Research) (IEC2 508/2022) on December 2 ^nd^ 2022. This study was conducted in accordance with the ethical principles outlined in the Declaration of Helsinki. The study participants were categorized into three groups after screening, and written informed consent was obtained from each participant before the focus group discussion. Participants were assured of their confidentiality and the voluntary nature of their involvement, meaning they were free to withdraw at any time without consequence.

### Author contribution

Conceptualization and methodology - AKG, NN, REL, VB, NB, VK, and MKS; formal analysis, AKG, NN, REL, and MKS; writing original draft - AKG, NN, REL, VB, NB, VK, and MKS; Review and editing - AKG, NN, REL, VB, NB, VK, and MKS; supervision, MKS, VK, NB, and VB

All authors have read, agreed, and reviewed the final version of the manuscript.

## Data Availability

All data underlying the results are available on Figshare. 1. Data Excell sheet for Young Adults Journey with Digital Fitness Tools. figshare. Dataset. https://doi.org/10.6084/m9.figshare.27249390.v2. ^15^ This study contains the following underlying data:
• Data Excell sheet for Young Adults Journey with Digital Fitness Tools Data are available under the terms of the CCO License. 2. Young adults journey with digital fitness tools-A qualitative study on use of fitness tracking device, figshare. Dataset. https://doi.org/10.6084/m9.figshare.27274944.v1. ^16^ This study contains the following extended data:
• Key questions in the moderator guide (Extended data (Moderator guide).docx) Data are available under the terms of the CCO License.

## References

[1] Strain T Flaxman S Guthold R : National, regional, and global trends in insufficient physical activity among adults from 2000 to 2022: a pooled analysis of 507 population-based surveys with 5· 7 million participants. *Lancet Glob. Health.* 2024 Aug 1;12(8):e1232–e1243. 10.1016/S2214-109X(24)00150-5 38942042 PMC11254784

[2] WHO reports: [Accessed on 24th July 2024]. Reference Source

[3] Lee IM Shiroma EJ Lobelo F : Effect of physical inactivity on major non-communicable diseases worldwide: an analysis of burden of disease and life expectancy. *Lancet.* 2012 Jul 21;380(9838):219–229. 10.1016/S0140-6736(12)61031-9 22818936 PMC3645500

[4] Min C Yoo DM Wee JH : Mortality and cause of death in physical activity and insufficient physical activity participants: A longitudinal follow-up study using a national health screening cohort. *BMC Public Health.* 2020 Dec;20:1. 10.1186/s12889-020-09564-x 32993602 PMC7526194

[5] Burford K Golaszewski NM Bartholomew J : “I shy away from them because they are very identifiable”: A qualitative study exploring user and non-user’s perceptions of wearable activity trackers. *Digit. Health.* 2021 Nov;7:20552076211054922. 10.1177/20552076211054922 34820134 PMC8606926

[6] Wilde LJ Ward G Sewell L : Apps and wearables for monitoring physical activity and sedentary behaviour: A qualitative systematic review protocol on barriers and facilitators. *Digit. Health.* 2018 May;4:2055207618776454. 10.1177/2055207618776454 29942637 PMC6016566

[7] Fabbrizio A Fucarino A Cantoia M : Smart devices for health and wellness applied to tele-exercise: an overview of new trends and technologies such as IoT and AI. *Healthcare.* 2023 Jun 20; Vol.11(12): p.1805. MDPI. 10.3390/healthcare11121805 37372922 PMC10298072

[8] Barkley JE Lepp A : Mobile phone use among college students is a sedentary leisure behavior which may interfere with exercise. *Comput. Hum. Behav.* 2016 Mar 1;56:29–33. 10.1016/j.chb.2015.11.001

[9] Lepp A Barkley JE Karpinski AC : The relationship between cell phone use, academic performance, anxiety, and satisfaction with life in college students. *Comput. Hum. Behav.* 2014 Feb 1;31:343–350. 10.1016/j.chb.2013.10.049

[10] Fennell C Barkley JE Lepp A : The relationship between cell phone use, physical activity, and sedentary behavior in adults aged 18–80. *Comput. Hum. Behav.* 2019 Jan 1;90:53–59. 10.1016/j.chb.2018.08.044

[11] Tong A Sainsbury P Craig J : Consolidated criteria for reporting qualitative research (COREQ): a 32-item checklist for interviews and focus groups. *Int. J. Qual. Health Care.* 2007 Dec 1;19(6):349–357. 10.1093/intqhc/mzm042 17872937

[12] Hu J He W Zhang J : Examining the impacts of fitness app features on user well-being. *Inf. Manag.* 2023 Jul 1;60(5):103796. 10.1016/j.im.2023.103796

[13] Voth EC Oelke ND Jung ME : A theory-based exercise app to enhance exercise adherence: a pilot study. *JMIR Mhealth Uhealth.* 2016 Jun 15;4(2):e4997. 10.2196/mhealth.4997 PMC4936794 27307134

[14] Tong HL Laranjo L : The use of social features in mobile health interventions to promote physical activity: a systematic review. *NPJ Digit. Med.* 2018 Sep 4;1(1):43. 10.1038/s41746-018-0051-3 31304323 PMC6550193

[15] Sinha MK : Data Excell sheet for Young Adults Journey with Digital Fitness Tools.Dataset. *figshare.* 2024. 10.6084/m9.figshare.27249390.v2 PMC11555328 39534658

[16] Sinha MK : Extended data (Moderator guide).docx.Dataset. *figshare.* 2024. 10.6084/m9.figshare.27274944.v1

