# Supplementary material for: Young Adults Journey with Digital Fitness Tools-A Qualitative Study on Use of Fitness Tracking Device
Source: F1000Res. 2024 Oct 29;13:1296. [Version 1] doi: 10.12688/f1000research.158037.1 (PMC11555328; doi:10.12688/f1000research.158037.1)
Supplement: Peer review history

# Reviewer report: Reviewer response for version 1

**DOI:** 10.5256/f1000research.173574.r337153

**Published:** 2024-11-11

**Authors:** Dr. Trapthi Kamath

**Affiliations:** 1 RV college of Physiotherapy, Bengaluru, Karnataka, India

This research investigates the experiences young adults with digital fitness tools mainly FGDs.By examining data  from young adults who currently use,have previously used or do not use  wearable fitness trackers.The research looks at how these devices influence physical activity habits,motivation and perceptions.The results indicate wearables can encourage physical activity through goal setting ,feedback and social interactions,such as device accuracy ,cost and technical issue for long term .The qualitative approach offers valuable insights into the complex experiences and attitudes of young adults toward wearable fitness tolls ,emphasizing the necessary designs that are user friendly and accesible.

 key insights- motivation,challenges,reach out, technological engagement.

 Strengthen of the study-diverse participant group

 areas of improvement-thematic consolidation

 Additionally add practical implications

Is the work clearly and accurately presented and does it cite the current literature?

Yes

If applicable, is the statistical analysis and its interpretation appropriate?

Yes

Are all the source data underlying the results available to ensure full reproducibility?

Yes

Is the study design appropriate and is the work technically sound?

Yes

Are the conclusions drawn adequately supported by the results?

Yes

Are sufficient details of methods and analysis provided to allow replication by others?

Yes

Reviewer Expertise:

neuro physiotherapy

I confirm that I have read this submission and believe that I have an appropriate level of expertise to confirm that it is of an acceptable scientific standard.

# Reviewer report: Reviewer response for version 1

**DOI:** 10.5256/f1000research.173574.r337150

**Published:** 2024-11-11

**Authors:** Aishwarya Nair

**Affiliations:** 1 Nitte Institute of Physiotherapy, NITTE (Deemed to be University),, Mangaluru, Karnataka, India

**SUMMARY**

 This study discusses the importance of physical activity and its impact on the young generation. As the current generation is perfectly synced with technology, they have a variety of applications at their disposal which allows them to track the levels of their physical activity. The study describes the complete protocol for selection of participants, the methods of FGD and the proceedings of the discussions. Clarity on the domains of the interview are provided clearly. The methodology is at par with the guideline issued for FGDs. A few clarifications are needed for the inclusion and exclusion criteria which has been described below. The details of the interview guide development is particularly well defined which is appreciated. The thematic diagram denoting the various data from the FGD is unique and attractive. It allows the reader to understand the study in a quick glance. It is better to avoid direct quotes from the participants as it introduces a certain casualness in the scientific document.   

**MAJOR REVISION-**

 INTRODUCTION

 1. A few studies that reported the reason for discontinuing the use of fitness can be added to the introduction. This will provide strong support for the need of this study.

 METHODOLOGY

 1. Please justify why individuals with recent injuries or fractures have been excluded especially since this study aims to understand their journey with the fitness apps. A recent journey may not negatively impact or hinder the past experience of using these fitness apps.

 DISCUSSION/CONCLUSION:

 1. Please add the strengths and limitations of this study with a note on scope for future studies.

**MINOR REVISION:**

 1. A complete grammar check can be done for the document.

Is the work clearly and accurately presented and does it cite the current literature?

Yes

If applicable, is the statistical analysis and its interpretation appropriate?

Yes

Are all the source data underlying the results available to ensure full reproducibility?

Yes

Is the study design appropriate and is the work technically sound?

Yes

Are the conclusions drawn adequately supported by the results?

Yes

Are sufficient details of methods and analysis provided to allow replication by others?

Yes

Reviewer Expertise:

Pulmonary rehabilitation, Fitness, Critical care physiotherapy, cardiac rehabilitation

I confirm that I have read this submission and believe that I have an appropriate level of expertise to confirm that it is of an acceptable scientific standard.
